# Estrogen receptor-beta sensitizes breast cancer cells to the anti-estrogenic actions of endoxifen

**DOI:** 10.1186/bcr2844

**Published:** 2011-03-10

**Authors:** Xianglin Wu, Malayannan Subramaniam, Sarah B Grygo, Zhifu Sun, Vivian Negron, Wilma L Lingle, Matthew P Goetz, James N Ingle, Thomas C Spelsberg, John R Hawse

**Affiliations:** 1 Department of Biochemistry and Molecular Biology, Mayo Clinic, 200 1stStreet SW, Rochester, MN 55905, USA; 2 Bioinformatics Core, Division of Biomedical Statistics and Informatics, Mayo Clinic, 200 1st Street SW, Rochester, MN 55905, USA; 3 Department of Laboratory Medicine and Pathology, Mayo Clinic, 200 1st Street SW, Rochester, MN 55905, USA; 4 Department of Oncology, Mayo Clinic, 200 1st Street SW, Rochester, MN 55905, USA

## Abstract

**Introduction:**

We have previously demonstrated that endoxifen is the most important tamoxifen metabolite responsible for eliciting the anti-estrogenic effects of this drug in breast cancer cells expressing estrogen receptor-alpha (ERα). However, the relevance of ERβ in mediating endoxifen action has yet to be explored. Here, we characterize the molecular actions of endoxifen in breast cancer cells expressing ERβ and examine its effectiveness as an anti-estrogenic agent in these cell lines.

**Methods:**

MCF7, Hs578T and U2OS cells were stably transfected with full-length ERβ. ERβ protein stability, dimer formation with ERα and expression of known ER target genes were characterized following endoxifen exposure. The ability of various endoxifen concentrations to block estrogen-induced proliferation of MCF7 parental and ERβ-expressing cells was determined. The global gene expression profiles of these two cell lines was monitored following estrogen and endoxifen exposure and biological pathway analysis of these data sets was conducted to identify altered cellular processes.

**Results:**

Our data demonstrate that endoxifen stabilizes ERβ protein, unlike its targeted degradation of ERα, and induces ERα/ERβ heterodimerization in a concentration dependent manner. Endoxifen is also shown to be a more potent inhibitor of estrogen target genes when ERβ is expressed. Additionally, low concentrations of endoxifen observed in tamoxifen treated patients with deficient CYP2D6 activity (20 to 40 nM) markedly inhibit estrogen-induced cell proliferation rates in the presence of ERβ, whereas much higher endoxifen concentrations are needed when ERβ is absent. Microarray analyses reveal substantial differences in the global gene expression profiles induced by endoxifen at low concentrations (40 nM) when comparing MCF7 cells which express ERβ to those that do not. These profiles implicate pathways related to cell proliferation and apoptosis in mediating endoxifen effectiveness at these lower concentrations.

**Conclusions:**

Taken together, these data demonstrate that the presence of ERβ enhances the sensitivity of breast cancer cells to the anti-estrogenic effects of endoxifen likely through the molecular actions of ERα/β heterodimers. These findings underscore the need to further elucidate the role of ERβ in the biology and treatment of breast cancer and suggest that the importance of pharmacologic variation in endoxifen concentrations may differ according to ERβ expression.

## Introduction

Each year, nearly 1.3 million women are diagnosed with breast cancer worldwide and about two-thirds of these individuals are determined to have hormone sensitive tumors based on the expression of estrogen receptor-alpha (ERα). Tamoxifen, a selective estrogen receptor modulator (SERM), remains an important therapeutic agent in the treatment of women with endocrine sensitive breast cancer as it is known to effectively inhibit the proliferation-inducing effects of 17β-estradiol (estrogen) in ERα positive breast tumor cells.

Like many drugs, tamoxifen is extensively metabolized in the body by the cytochrome P450 enzyme system resulting in the production of three primary metabolites; 4-hydroxytamoxifen (4HT), N-desmethyl-tamoxifen (NDT) and endoxifen [1-3]. Recent reports have demonstrated that steady state circulating levels of tamoxifen, 4HT, and NDT in women receiving the standard dose of tamoxifen therapy (20 mg/day) are 300 nM, 7 nM, and 700 nM, respectively [4]. However, plasma endoxifen concentrations are highly variable, ranging from 5 to 180 nM, due to the activity of the cytochrome P450 2D6 (CYP2D6) mediated oxidation of NDT [3]. Prospective studies have demonstrated that genetic CYP2D6 polymorphisms, and drugs, which reduce or abrogate CYP2D6 enzyme activity, significantly decrease endoxifen plasma concentrations [3-5]. These findings encouraged investigators to examine the hypothesis that CYP2D6 genotype status, and thus endoxifen concentrations, would affect clinical outcome in women treated with tamoxifen for their breast cancer. Although some controversy remains, the majority of the reports indicate a relationship between CYP2D6-related low levels of endoxifen and poor outcomes [6-15]. Past studies from this laboratory support these clinical findings as we have demonstrated that endoxifen is the most potent tamoxifen metabolite responsible for inhibiting estrogen induced gene expression changes and proliferation rates in ERα positive breast cancer cells at clinically relevant concentrations [16]. At this time, the clinical development of endoxifen is ongoing, with NCI supported phase I studies of endoxifen hydrochloride set to commence in early 2011 at both the Mayo Clinic and NCI.

Tamoxifen and its metabolites are known to function by blocking the effects of estrogen, a steroid hormone that binds to, and activates, two main ER isoforms, ERα and ERβ. The role of ERα in breast cancer has been studied extensively for years, and its protein expression remains the most important biomarker in the treatment of this disease. However, the potential functions of ERβ in the progression and treatment of breast cancer have largely remained a mystery. *In vitro *studies have revealed that the actions of these two receptors are drastically different at the level of gene expression, both in response to estrogen and anti-estrogens [17-23]. Numerous reports have demonstrated that exposure of ERα expressing breast cancer cells to estrogen results in increased rates of proliferation while more recent studies have suggested that expression of ERβ alone [21,24,25], or in combination with ERα [26-28], inhibits cell proliferation following estrogen exposure.

The summation of these *in vitro *studies suggests that ERβ may function as a tumor suppressor. A number of clinical studies have revealed that the presence of ERβ protein in breast tumors correlates with improved rates of recurrence, disease-free survival and overall survival [29-38] while others indicate little correlation [39-41] or even worse prognosis [42,43]. Additional studies have suggested that the expression of ERβ in breast tumors increases the effectiveness of tamoxifen therapy [44-46] and one report found that 47% of breast tumors classified as ERα negative express ERβ [33]. These observations highlight the need to further define the relevance of ERβ in breast cancer progression and treatment.

Based on the foregoing, the objective of the present study was to determine the role of ERβ in mediating endoxifen action in breast cancer cells. The results of this study demonstrate that ERβ enhances the anti-estrogenic effects of endoxifen in breast cancer cells likely through the actions of ERα/β heterodimers, and suggest that the achievement of higher endoxifen concentration may not be necessary in patients whose tumors express ERβ and that these same patients may benefit from tamoxifen therapy regardless of their CYP2D6 genotype.

## Materials and methods

### Cell culture, chemicals and reagents

MCF7 cells were generously provided by Dr. Robert Clarke (Georgetown University) and were cultured in phenol red-free Dulbecco's modified Eagle's medium/F12 (DMEM/F12) medium containing 10% (v/v) fetal bovine serum (FBS) and 1% (v/v) antibiotic-antimycotic (AA) solution in a humidified 37°C incubator with 5% CO_2_. MCF7 cells stably expressing ERβ were generated using an S-tagged-Flag-tagged ERβ expression construct (pIRES2-EGFP) developed in our laboratory. Individual MCF7-ERβ clones were isolated following selection with 300 μL/mL G418. Doxycycline inducible Hs578T-ERβ, U2OS-ERβ and U2OS-ERα/β cells lines were cultured as previously described [19-21]. All cell treatments were conducted in phenol red-free DMEM/F12 medium containing 10% triple charcoal stripped FBS. 17β-estradiol and doxycyline were purchased from Sigma Aldrich (St. Louis, MO, USA). PPT (propyl pyrazole triol) and DPN (diarylpropionitrile) were purchased from Tocris Biosciences Inc. (Baldwin, MO, USA). (Z)-endoxifen was synthesized by Dr. Abdul Fauq (Mayo Clinic, Jacksonville, FL, USA).

### Antibodies

The polyclonal ERβ specific antibody utilized in this study was developed by this laboratory. Briefly, a protein fragment of ERβ spanning amino acids 1 to 140 was cloned into the pGEX-5X-3 vector (Life Technologies, Carlsbad, CA, USA) and expressed in DH5α bacterial cells. Purified ERβ protein was immunized in rabbits by Cocalico Biologicals Inc (Reamstown, PA, USA). ERβ specific antibodies were purified from serum using an affinity purification column containing the ERβ protein fragment. We have extensively characterized this antibody through Western blotting, immunoprecipitation, immunohistochemistry and immunoflourescence using multiple cell model systems which express either no ERs, ERα alone, ERβ alone or a combination of ERα and ERβ, to ensure its specificity for the β isoform. These data have revealed that this antibody is highly sensitive for the detection of ERβ and exhibits no cross-reaction with ERα or other proteins (data not shown). ERα (H-20) antibody was purchased from Santa Cruz Biotechnology (Santa Cruz, CA, USA). Flag (M2) and α-Tubulin (DM 1A) antibodies were purchased from Sigma Aldrich.

### Western blotting

All cell lysates were harvested using NETN buffer (150 mM NaCl, 1 mM EDTA, 20 mM Tris (pH 8.0), 0.5% Nonidet P-40) and insoluble material was pelleted. Protein concentrations were determined using Bradford Reagent and equal amounts of cell lysate were separated by SDS-PAGE. Proteins were transferred to PVDF membranes, probed with primary and secondary antibodies and visualized using enhanced chemiluminescence (Amersham Biosciences, Piscataway, NJ, USA).

### Immunofluorescent staining analysis

MCF7-ERβ cells were plated on cover slides at approximately 50% confluence and fixed with cold methanol for one hour followed by permeabilization with 0.1% Triton X-100 for five minutes on ice. Slides were pre-incubated in 5% goat serum for one hour followed by exposure to Flag antibody for another hour. Slides were washed three times with 1× PBS and subsequently incubated with Rhodamine-labeled anti-mouse secondary antibody (Sigma-Aldrich) for 30 minutes at room temperature. Nuclei were simultaneously stained with DAPI (Sigma-Aldrich). Immunofluorescent detection was conducted using a Zeiss Laser Scanning Microscope 510 (Carl Zeiss, Jena, Germany)

### Luciferase assays

MCF7-ERβ cells were plated in 12-well tissue culture plates at approximately 70% confluence and subsequently transfected in triplicate with 250 ng per well of the ERE-TK-luciferase reporter construct using Fugene 6 (Roche Applied Science, Indianapolis, IN, USA). Following transfection, cells were treated as indicated for 24 h. Cells were lysed in 1× Passive Lysis Buffer (Promega, Madison, WI, USA) and equal amounts of extract were assayed for luciferase activity.

### Co-Immunoprecipitation assays

MCF7-ERβ or U2OS-ERα/β cells were plated at a density of approximately 50% in 100 mm tissue culture plates. U2OS cells were treated with doxycycline as previously described to induce expression of ERα and ERβ [20] and subsequently exposed to indicated concentrations of endoxifen. Following 24 hours of incubation, cells were washed twice with PBS and lysed in NETN buffer. Equal amounts of cell lysates were immunoprecipitated at 4°C overnight using 1 μg of either Flag or ERβ antibody. Protein complexes were purified using protein G beads, separated by SDS-PAGE, transferred to PVDF membranes and blocked in 5% milk overnight. Western blotting was performed using indicated antibodies as described above.

### Real-time reverse transcriptase polymerase chain reaction

MCF7 and MCF7-ERβ cells were plated in 12-well tissue-culture plates and treated in triplicate as indicated for 24 hours. Total RNA was isolated using Trizol reagent (Invitrogen) and 500 ng was reverse transcribed using the iScript™ cDNA Synthesis Kit (Bio-Rad). Real-time PCR was performed in triplicate using a Bio-Rad iCycler (Hercules, CA, USA) and a PerfeCTa™ SYBR Green Fast Mix™ for iQ real-time PCR kit (Quanta Biosciences, Gaithersburg, MD, USA) as specified by the manufacturer. Quantitation of the PCR results were calculated based on the threshold cycle (C_t_) and were normalized using TATA Binding Protein as a control. All PCR primers were designed using Primer3 software [47] and were purchased from Integrated DNA Technologies (Coralville, IA, USA). Primer sequences are provided in Additional file 1.

### Cell proliferation assays

MCF7 and MCF7-ERβ cells were grown in 10% triple charcoal-stripped serum-containing medium for three days and subsequently plated at a density of 2,000 cells per well in 96-well tissue culture plates. Cells were treated with vehicle, 1 nM estrogen or 1 nM estrogen plus increasing concentrations of endoxifen (20 to 1,000 nM) for eight days. Culture medium and treatments were replaced every other day. Proliferation rates were determined using a CellTiter-Glo Luminescent Cell Viability kit (Promega).

### Illumina microarray analysis

Changes in the gene expression profiles of MCF7 and MCF7-ERβ cells elicited by either 1 nM estrogen alone or estrogen plus 40 nM endoxifen were determined using the Illumina HumanHT-12 expression BeadChip platform to screen more than 27,000 annotated genes represented by 48,804 probes by Mayo Clinic's Advanced Genomics Technology Center (Rochester, MN, USA). Data were processed using BeadStudio Version 3.1 and normalized using the fastlo function [48] implemented in the statistical package R. Data were filtered to exclude probes (referred to as genes throughout) whose expression was at or below background levels as determined by detection *P*-values (≥0.05). Pair-wise comparisons were made to identify differentially expressed genes using Linear Models for Microarray Data (LIMMA). Genes were determined to be significantly regulated if their differential *P*-value was < 0.05 between groups. Fold changes were calculated by raising 2 to the power of mean difference (log 2 scale) between the treatment groups and controls. The normalized and raw microarray data presented here are available in the Gene Expression Omnibus [49] under the accession number: [GEO:GSE27375].

### Biological pathway analysis

Genes determined to be significantly regulated by the addition of 40 nM endoxifen relative to estrogen alone in both parental and ERβ expressing MCF7 cells were further analyzed using MetaCore software to identify differences in biological pathways altered between the two cell lines. Genes with differential expression *P*-values < 0.05 from each comparison were used as focus genes and a hypergeometric test was applied to each of over 600 canonical pathways. Enriched pathways with *P*-values < 0.05 were suggested to be significantly regulated by the addition of 40 nM endoxifen within each cell line. Adjustment for multiple comparisons was conducted using a false discovery rate of 0.25.

## Results

### Development and characterization of MCF7-ERβ cell lines

**Figure 1:**
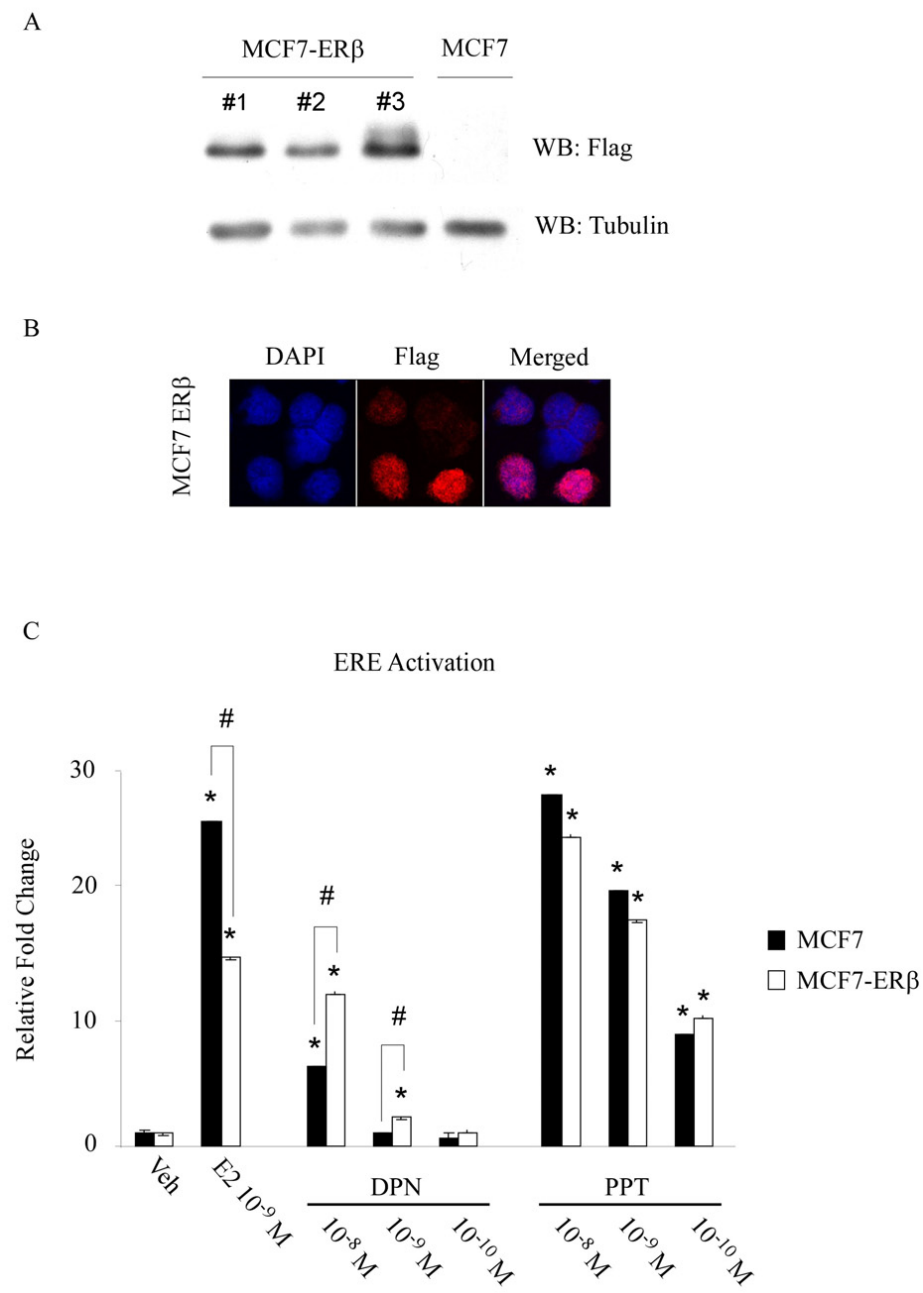
**Characterization of MCF7-ERβ cell lines**. **(A) **Western blot (WB) analysis demonstrating expression of ERβ in three independent clonal MCF7 cell lines. **(B) **Immunoflourescence depicting nuclear localization of ERβ protein in stably expressing MCF7 cell lines. **(C) **Luciferase assays demonstrating transcriptional activation of a transiently transfected ERE by either ERα or ERβ in parental and MCF7-ERβ cell lines using estrogen, the ERα specific agonist PPT or the ERβ specific agonist DPN. * denotes significance at the *P *< 0.05 level (ANOVA) compared with vehicle controls while # denotes significance for a given treatment between the two cell lines.

**Figure 2:**
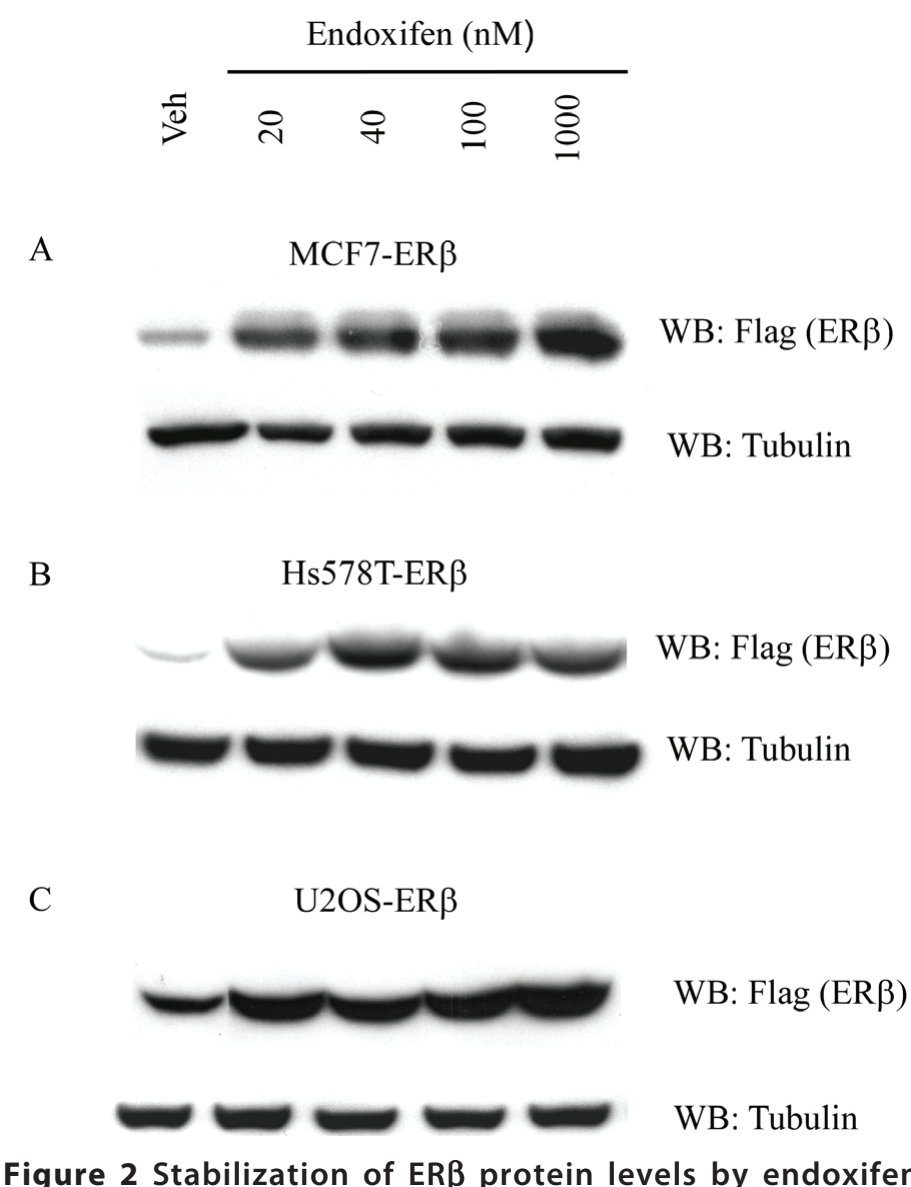
**Stabilization of ERβ protein levels by endoxifen**. Western blot (WB) analysis of ERβ protein levels in MCF7-ERβ **(A)**, Hs578T-ERβ **(B) **and U2OS-ERβ **(C) **cells treated with indicated concentrations of endoxifen or vehicle for 24 hours. Tubulin levels are shown as protein loading controls.

**Figure 3:**
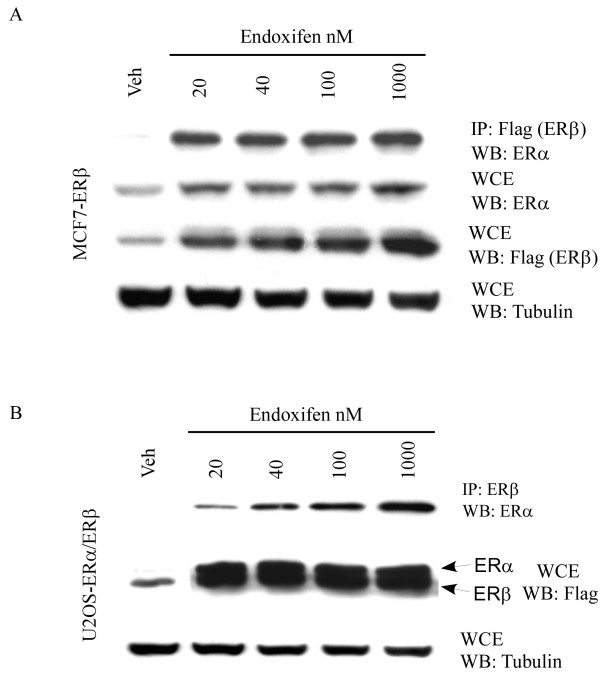
**Endoxifen induces ERα/β heterodimer formation**. MCF7-ERβ **(A) **or U2OS-ERα/β **(B) **cells were treated with indicated concentrations of endoxifen or vehicle for 24 hours. Equal amounts of cell lysates were immunoprecipitated with an ERβ specific antibody. Immunoprecipitated protein (IP) complexes were separated by SDS-PAGE and Western blotting (WB) was performed using an ERα specific antibody. Non-immunoprecipitated ERα and ERβ protein levels were also determined by Western blotting in whole cell extracts (WCE) following endoxifen treatment. Tubulin levels are shown as protein loading controls.

**Figure 4:**
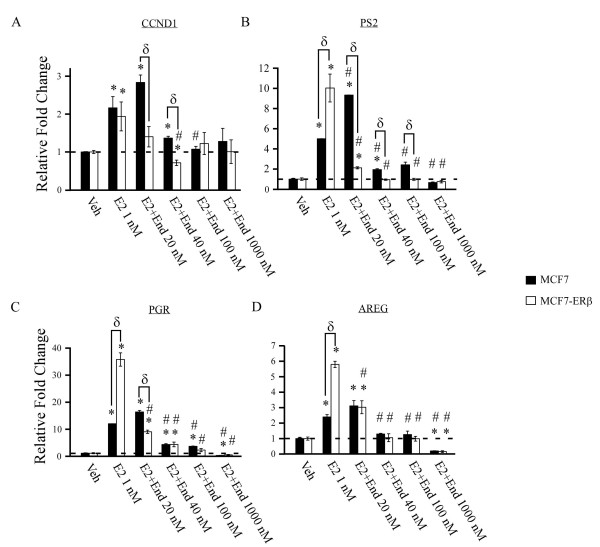
**Repression of known ER target genes by endoxifen is enhanced in ERβ expressing cells**. Parental MCF7 and MCF7-ERβ cells were treated as indicated for 24 hours. Real-time RT-PCR analysis was performed to detect expression levels of **(A) **cyclin D1 (CCND1), **(B) **PS2, **(C) **progesterone receptor (PR) and **(D) **amphiregulin (AREG). * denotes significance at the *P *< 0.05 level (ANOVA) compared to vehicle, # compared to estrogen treatment and δ for a given treatment between the two cell lines.

In order to determine the effects of ERβ expression on the actions of endoxifen in breast cancer, we first developed MCF7 cell lines stably expressing this receptor. MCF7 cells were chosen for this study since they are the most well characterized ERα positive breast cancer cell line with regard to estrogen regulated gene expression and proliferation. As shown in Additional file 2, the parental MCF7 cell line used throughout this study was confirmed to be ERβ negative by both real-time PCR and Western blotting and these data are shown relative to the expression of ERβ mRNA and protein in one of our over-expressing cell lines. Multiple MCF7-ERβ clonal cell lines were developed by expressing S and Flag-tagged full-length ERβ followed by selection with G418. All cell lines were screened for ERβ protein expression by Western blotting and three representative lines are shown in Figure 1A relative to parental cells. While all of the data presented in Figures 1, 2, 3, 4 were confirmed in multiple MCF7-ERβ clones, the data collected from clone number 3 were chosen for representation in these figures due to its robust expression of ERβ. Immunofluorescent staining using a flag antibody was utilized to verify ERβ positivity and cellular localization. The results demonstrate that ERβ is expressed and localized in the nucleus of MCF7-ERβ cells (Figure 1B). ERβ functionality was investigated using an estrogen response element (ERE) transcriptional assay involving an ERE-TK-luciferase reporter construct. The construct was transfected into parental and ERβ expressing MCF7 cells followed by treatment with either estrogen, the ERα specific agonist PPT or the ERβ specific agonist DPN. As shown in Figure 1C, estrogen significantly induced ERE activity in both MCF7 parental and ERβ expressing cells. Interestingly, estrogen induction of the ERE was significantly lower in ERβ expressing cells possibly due to the reported inhibitory actions of ERβ on ERα transcriptional activity. The ERβ specific agonist, DPN, resulted in significant activation of the ERE reporter construct in cells expressing ERβ (Figure 1C). ERE activation in parental MCF7 cells by 10^-8 ^M DPN is explained by its non-specific interactions with ERα at high concentrations [50]. As expected, all concentrations of the ERα specific ligand, PPT, resulted in identical ERE activation regardless of ERβ expression (Figure 1C). These data demonstrate that our newly developed MCF7-ERβ cell lines express intact and functional ERβ protein.

### ERβ protein levels are stabilized by endoxifen

Since we have previously demonstrated that endoxifen exposure results in rapid turnover of ERα protein in multiple cell types through proteasomal degradation [16], it was of interest to determine the effects of endoxifen on ERβ protein levels. Unlike that of ERα, endoxifen exposure resulted in stabilization of ERβ protein in MCF7-ERβ cells in a concentration dependent manner (Figure 2A). These results were confirmed in Hs578T breast cancer cells and U2OS osteosarcoma cells stably expressing ERβ (Figure 2B, C).

### Endoxifen induces ERα/β heterodimer formation

Given that ERβ protein levels are stabilized by endoxifen and that ERβ interacts with ERα, we next sought to determine if endoxifen exposure resulted in ERα/β heterodimer formation. Immunoprecipitation assays demonstrate that endoxifen induces ERα/β heterodimer formation in MCF7-ERβ cells which results in the stabilization and accumulation of ERα protein levels (Figure 3A). These studies were conducted in U2OS cells stably expressing both ER isoforms and similar results were observed (Figure 3B). The results of these studies demonstrate that exposure of cells which express both ER isoforms to endoxifen results in stabilization and accumulation of both ERα and ERβ protein likely due to its induction of heterodimer formation. It is speculated that ERα and ERβ homodimer formation likely occurs to some degree as well.

### Endoxifen's ability to inhibit estrogen induced gene expression and proliferation is enhanced by ERβ

Our laboratory previously characterized the inhibition of estrogen induced gene expression and proliferation by endoxifen in MCF7 cells [16]. In order to determine the effects of ERβ expression on the anti-estrogenic actions of endoxifen, we next compared the ability of endoxifen to inhibit estrogen induction of known ERα target genes in parental and ERβ expressing MCF7 cells. The expression levels of cyclinD1, PS2, progesterone receptor and amphiregulin were monitored in both cell lines by real-time PCR following treatment with estrogen alone or estrogen plus increasing concentrations of endoxifen. In contrast to the ERE data presented in Figure 1, estrogen treatment further stimulated the expression of three of the four genes (PS2, PGR and AREG) in ERβ expressing cells relative to the parental cell line (Figure 4A-D). Interestingly, low concentrations of endoxifen (20 nM) significantly inhibited the estrogen induction of three of the four genes (CCND1, PS2 and PGR) only in ERβ expressing cells while higher endoxifen concentrations (100 to 1,000 nM) resulted in similar patterns of gene expression between the two cell lines (Figure 4A-D). These data suggest that expression of ERβ, in ERα positive breast cancer cells, enhances the anti-estrogenic properties of endoxifen.

**Figure 5:**
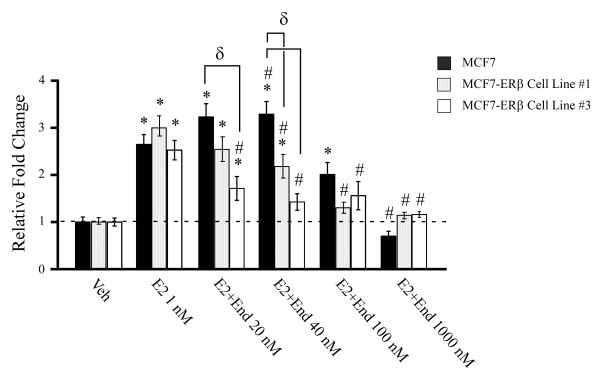
**Low concentrations of endoxifen inhibit estrogen induced proliferation of MCF7-ERβ cells**. Parental and MCF7-ERβ cells (cell lines #1 and #3) were treated as indicated for eight days and cell proliferation rates were analyzed. Graphs depict fold change from vehicle treated cells. * denotes significance at the *P *< 0.05 level (ANOVA) compared to vehicle controls, # compared to estrogen treated cells and δ for a given treatment between the two cell lines.

To confirm these data, and to determine if expression of ERβ enhances the ability of endoxifen to suppress estrogen induced cell growth, proliferation assays were performed. Two independent ERβ expressing cell lines are shown for these studies to ensure that the results are due to expression of ERβ and not clonal variation. Induction of cell proliferation following estrogen treatment was identical between parental and ERβ expressing MCF7 cells (Figure 5). Similar to the gene expression data presented in Figure 4, low concentrations of endoxifen (20 to 40 nM) significantly inhibited estrogen induced growth of MCF7-ERβ cells but not parental MCF7 cells (Figure 5). In fact, 100 to 1,000 nM concentrations of endoxifen were required to completely block estrogen induced growth of parental MCF7 cells while 20 to 40 nM endoxifen concentrations were essentially as effective in ERβ positive cells (Figure 5). These studies confirm that ERβ expression sensitizes breast cancer cells to the anti-estrogenic actions of endoxifen.

### ERβ expression results in unique gene expression patterns following estrogen and endoxifen exposure

**Figure 6:**
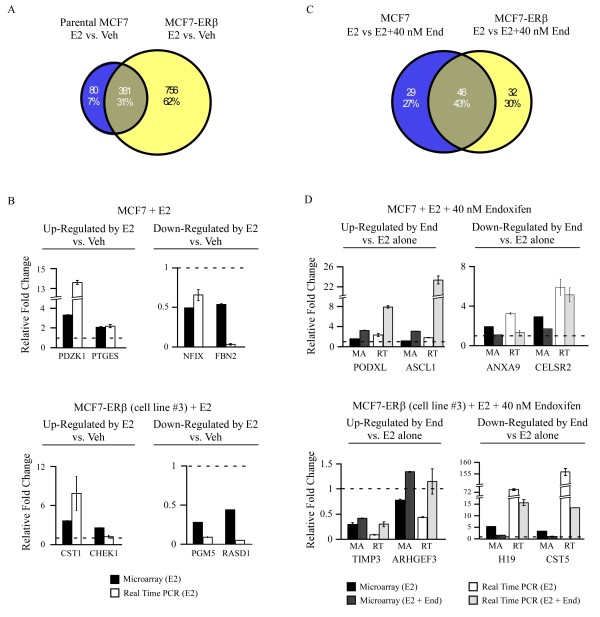
**Microarray analysis of estrogen and estrogen plus endoxifen treatment in parental and MCF7-ERβ cells**. (**A and C**) Venn diagrams indicating the number of genes whose expression levels were significantly altered by at least 1.5-fold in response to 24-hour treatments of 1 nM estrogen in MCF7 or MCF7-ERβ cells (**A**) or 1 nM estrogen + 40 nM endoxifen relative to estrogen treatment alone (**C**). (**B and D**) Real-time PCR confirmation of selected genes whose expression levels were either increased or decreased by the addition of estrogen in MCF7 or MCF7-ERβ cells (**B**) or 1 nM estrogen + 40 nM endoxifen relative to estrogen treatment alone (**D**). The fold changes of each gene as detected by microarray analysis are shown for comparison purposes and all data have been normalized to vehicle controls (dotted line).

In an effort to determine the mechanisms by which low concentrations of endoxifen effectively block estrogen induced growth of ERβ expressing cells, but not parental MCF7 cells, the global gene expression profiles were examined in these two cell lines following treatment with estrogen alone or estrogen plus 40 nM endoxifen. The 40 nM endoxifen concentration was chosen since it resulted in the largest differences in proliferation rates between parental and MCF7-ERβ cells. Microarray analysis revealed that estrogen treatment significantly altered the expression of 461 genes in parental MCF7 cells using a fold change cutoff of 1.5 (Figure 6A). Of these genes, 211 exhibited increased expression while 251 exhibited decreased expression. Nearly 2.5 times as many genes were determined to be significantly regulated in the MCF7-ERβ cell line using the same statistical and fold change cutoff parameters. Specifically, 1,137 genes were differentially expressed following estrogen treatment of which 604 were increased and 535 were decreased (Figure 6A). Comparison of these two data sets revealed that 381 (31%) were commonly regulated between the two cell lines, while only 80 (7%) were unique to the parental cell line and 756 (62%) were unique to the ERβ line (Figure 6A). A list of these genes and their detected fold changes is provided in Additional file 3. Two genes exhibiting increased expression and two genes exhibiting decreased expression in response to estrogen treatment were randomly selected for each cell line and confirmed by real-time PCR. The relative expression levels for these genes as determined by both microarray and real-time PCR are shown in Figure 6B.

We next compared the microarray results of parental and MCF7-ERβ expressing cells treated with estrogen plus 40 nM endoxifen to estrogen treatment alone. Using the same selection criteria as above, significantly fewer genes were determined to be regulated by this dose of endoxifen. In the parental cell line, the expression levels of 75 genes were altered by at least 1.5-fold by the addition of endoxifen of which 44 were increased and 31 were decreased (Figure 6C). Similar results were observed in the MCF7-ERβ cell line in that a total of 78 genes were differentially regulated in response to endoxifen with 37 exhibiting increased expression and 41 exhibiting decreased expression (Figure 6C). Comparison of these two data sets indicated that 46 genes (43%) were commonly regulated in both cell lines while 29 (27%) were unique to the parental cells and 32 (30%) were unique to the ERβ expressing cells (Figure 6C). A list of these genes and their detected fold changes is provided in Additional file 4. As above, two genes exhibiting increased expression and two genes exhibiting decreased expression in response to endoxifen treatment were randomly selected for each cell line and confirmed by real-time PCR. The relative expression levels of these genes as determined by both microarray and real-time PCR following estrogen treatment alone and estrogen plus endoxifen are shown in Figure 6D.

**Figure 7:**
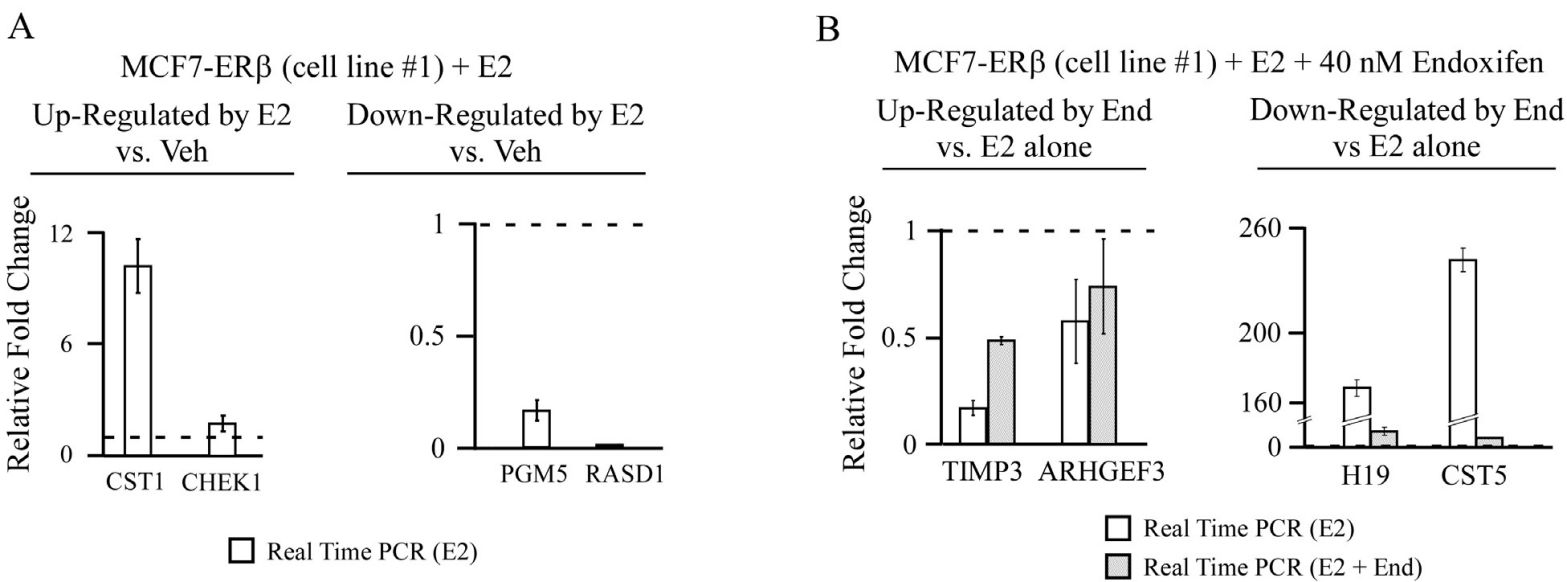
**Confirmation of microarray data in a second ERβ expressing MCF7 cell line**. **(A and B) **Real-time PCR confirmations were also carried out in a second ERβ cell line (#1) to ensure that the detected gene expression changes were due to the presence of ERβ and were not a result of potential clonal variation between cell lines. Relative fold changes of genes determined to be regulated by 1 nM estrogen alone (**A**) or by 1 nM estrogen + 40 nM endoxifen relative to estrogen treatment alone (**B**) are shown following normalization to vehicle controls (dotted line). The same trends in gene expression were detected in response to both estrogen and endoxifen in this second ERβ expressing cell line.

As with the proliferation data, to ensure that the detected gene expression differences in response to estrogen and endoxifen were truly due to the presence of ERβ and not a result of clonal variation, the confirmation of gene expression studies were also carried out in a second ERβ cell line (#1). These results revealed the same trends in gene expression elicited in response to both estrogen and endoxifen (Figure 7A, B) and suggest that these differences are in fact a result of ERβ expression and not due to clonal variation between cell lines.

### Pathway analysis identifies specific biological processes that are uniquely regulated by endoxifen in MCF7-ERβ cells

Pathway analysis was performed on the gene lists generated from both the parental and MCF7-ERβ cells treated with estrogen plus 40 nM endoxifen relative to estrogen alone. For this analysis, all genes whose expression levels were significantly altered (*P *< 0.05) by the addition of endoxifen and whose fold changes were >2 standard deviations away from all genes kept in the analysis (approximately 1.2-fold) were utilized. This analysis identified 13 pathways in the parental cell line and 12 pathways in the MCF7-ERβ cell line which had significant enrichment of genes based on a *P *< 0.05. While none of these pathways passed a false discovery rate threshold of 0.25 after adjusting for multiple comparisons likely due to relatively small numbers of genes, it is interesting to note that biological pathways involving ERα cell cycle regulation and cell migration were only affected by endoxifen in breast cancer cells expressing ERβ (Table 1). As with the gene expression data, many of the identified pathways were unique to either the parental or ERβ expressing cell lines (identified by an asterisk) lending further support to the differential effects of endoxifen as a result of ER isoform specific expression (Table 1).

**Table 1:** Biological pathways regulated by estrogen plus 40 nM endoxifen relative to estrogen treatment alone

	MCF7 Parental Cells		
Pathway #	Pathway Name	*P*-Value	# Genes
1*	Immune response_Antiviral actions of Interferons	0.0008	6/20
2*	Immune response_IFN gamma signaling pathway	0.0177	6/36
3	Regulation of lipid metabolism_Regulation of fatty acid synthase activity	0.0182	3/10
4	Neurodisease_Parkin disorder under Parkinson disease	0.0191	4/18
5*	Regulation of lipid metabolism_Regulation of acetyl-CoA carboxylase 1	0.0195	2/4
6*	Cholesterol Biosynthesis	0.0276	4/20
7*	Niacin-HDL metabolism	0.0305	3/12
8*	Cytoskeleton remodeling_Thyroliberin in cytoskeleton remodeling	0.0305	3/12
9	CFTR-dependent regulation of ion channels in Airway Epithelium	0.0326	4/21
10	Immune response_IL-27 signaling pathway	0.0380	3/13
11*	Development_A1 receptor signaling	0.0440	4/23
12*	Neurophysiological process_PGE2-induced pain processing	0.0450	2/6
13*	Immune response_Th1 and Th2 cell differentiation	0.0463	3/14
	**MCF7-ERβ Cells**		
1*	wtCFTR and deltaF508 traffic/Membrane expression (norm and CF)	0.0026	5/16
2	Regulation of lipid metabolism_Regulation of fatty acid synthase activity	0.0026	4/10
3	Immune response_IL-27 signaling pathway	0.0077	4/13
4*	Blood coagulation_Blood coagulation	0.0132	4/15
5*	Cell cycle_ERα regulation of G1/S transition	0.0167	5/24
6*	Globo-(isoglobo) series GSL Metabolism	0.0228	3/10
7	Neurodisease_Parkin disorder under Parkinson disease	0.0254	4/18
8*	ERα action on cytoskeleton remodeling and cell migration	0.0299	3/11
9*	Transcription_Ligand-Dependent Transcription of Retinoid-Target genes	0.0306	4/19
10*	ENaC regulation in airways (normal and CF)	0.0364	4/20
11	CFTR-dependent regulation of ion channels in Airway Epithelium	0.0427	4/21
12*	wtCFTR and delta508-CFTR traffic/Generic schema (norm and CF)	0.0463	5/31

## Discussion

### Endocrine sensitive breast cancer

The large majority of breast cancer patients display tumors that are estrogen dependent based on the expression of ERα. Deprivation of estrogen signaling, most commonly through the use of tamoxifen in the adjuvant setting, typically results in tumor regression. However, the use of ERα alone as an indicator of responsiveness to anti-estrogens is far from perfect as about one-half of ERα positive tumors do not respond to tamoxifen therapy and about 10% of ERα negative tumors do respond. These studies demonstrate that other estrogen and anti-estrogen receptors and/or signaling pathways may be involved in mediating the responsiveness of endocrine sensitive tumors to hormonal agents. Following the discovery of ERβ, investigators have sought to uncover the role that this protein may play in mediating breast cancer progression and treatment. Here, we demonstrate that expression of ERβ in ERα positive MCF7 cells significantly enhances the anti-estrogenic effects of endoxifen. This study provides evidence that endoxifen stabilizes ERβ protein levels and induces ERα/β heterodimer formation which results in differential gene expression patterns. Perhaps most importantly, our studies reveal that even low concentrations of endoxifen, mimicking that of poor and intermediate CYP2D6 metabolizers, results in repression of estrogen induced breast cancer proliferation in cells expressing ERβ, but not in those that express only ERα.

### Role of ERβ in breast cancer

ERβ is known to be expressed in normal breast epithelial cells and several studies have demonstrated that ERβ expression levels are suppressed in many breast cancers [51-54]. However, re-expression of ERβ in ER negative breast cancer cells has been shown to reduce both basal and estrogen induced proliferation rates [21,24,25]. Expression of ERβ in ERα positive breast cancer cells also results in suppression of proliferation following estrogen exposure [26-28]. Furthermore, ERβ expression has been shown to increase the effectiveness of high concentrations of SERMs such as 4HT, raloxifen and fulvestrant [28,55]*in vitro*. While the latter studies did not analyze endoxifen, nor did they utilize clinically relevant concentrations of 4HT (plasma concentrations are less than 10 nM in patients receiving 20 mg/day), they do further implicate a role for ERβ in mediating anti-estrogenic activities.

It is possible that the increased effectiveness of endoxifen in ERβ expressing MCF7 cells is related to the molecular actions of ERα/β heterodimers since we demonstrate that heterodimer formation is induced in a concentration dependent manner following endoxifen exposure. Indeed, the global gene expression changes induced by estrogen and anti-estrogens are known to be different in cells expressing both estrogen receptors relative to cells expressing only ERα or ERβ [20,21,56]. The results of the present study also demonstrate that both estrogen and endoxifen regulate unique subsets of genes in MCF7 cells expressing both receptors relative to cells expressing ERα alone. Biological pathway analysis of endoxifen regulated genes revealed that the majority of altered pathways are unique to either the parental or ERβ expressing cell lines. Pathways involving ERα mediated cell cycle regulation and cell migration were only affected in the presence of ERβ suggesting that the increased effectiveness of endoxifen may be through the inhibition of ERα activity by ERβ.

### Compounds which induce ER heterodimer formation as therapeutic drugs

The ability of specific compounds to induce ER heterodimer formation is of significant importance since these two receptors are often expressed in the same cells of many different tissues, including breast tumors, and since different dimer pairs have distinct genomic targets [57]. Of particular interest is the observation that genistein, a compound originally thought to contribute to decreased breast cancer risk, specifically induces ERα homodimerization and transcriptional activity [58]. This observation correlates well with more recent studies demonstrating that genistein is not effective in the prevention of breast cancer [59,60]. Conversely, liquiritigenin is a highly selective ERβ agonist which does not stimulate ERα positive tumor formation [61] or ERα homodimerization [58] suggesting that it may serve as a suppressor of proliferation in ERβ expressing cells. The summation of these studies indicates that the identification of compounds which can specifically induce and/or activate ERα/β heterodimers or ERβ homodimers may have therapeutic potential. Endoxifen may serve as such a compound since it stabilizes ERβ protein levels and induces heterodimer formation in cells expressing both ER isoforms while simultaneously degrading ERα protein in ERβ negative cells [16].

### Impact of ERβ positivity and increased endoxifen effectiveness

The identification of increased endoxifen effectiveness as an anti-estrogenic agent in the setting of ERβ is of significant clinical importance due to the fact that ERβ expression is reported to exist in approximately 75% of invasive breast cancers [33,36,37,42,62-64] and in a subset of tumors which are ERα negative [41,45,65]. The majority of reports suggest that the presence of ERβ in breast tumors correlates with improved rates of recurrence, disease-free survival and overall survival [29-38]; however, others indicate little correlation [39-41]. A few recent clinical studies have revealed that ERβ increases the effectiveness of tamoxifen therapy for breast cancer [44-46]. Given that endoxifen is being developed for the treatment of ER positive breast cancer, future studies should evaluate the association between ERβ expression and the activity of endoxifen in human breast tumors.

## Conclusions

The present data indicate that ERβ enhances the anti-estrogenic actions of endoxifen in breast cancer cells. These data correlate well with the clinical studies demonstrating increased benefit from tamoxifen therapy in those patients whose tumors are ERβ positive and suggest that this benefit may be through the actions of endoxifen. The ability of low endoxifen concentrations to significantly inhibit estrogen induced gene expression and proliferation in ERβ expressing breast cancer cells also suggests that benefits from tamoxifen therapy may still be observed in patients characterized as poor metabolizers based on CYP2D6 genotype if their tumors are ERβ positive. Finally, these studies highlight the need to further investigate the role of ERβ in breast cancer, both as a prognostic and predictive factor, and lend additional support to the development of endoxifen as a novel therapeutic for the treatment of endocrine sensitive breast tumors.

## Abbreviations

4HT: 4-hydroxy-tamoxifen; 17β-estrodiol: estrogen; AA: antibiotic/antimycotic; CYP2D6: cytochrome P450 2D6; DMEM/F12: Dulbecco's Modified Eagle's Medium/F12; DPN: diarylpropionitrile; ER: estrogen receptor; ERα: estrogen receptor-alpha; ERβ: estrogen receptor-beta; ERE: estrogen response element; FBS: fetal bovine serum; G418: geneticin; NCI: National Cancer Institute; NDT: N-desmethyl-tamoxifen; PPT: propyl pyrazole triol; RT-PCR: real-time polymerase chain reaction; SDS-PAGE: sodium dodecyl sulfate polyacrylamide gel electrophoresis; SERM: selective estrogen receptor modulator.

## Competing interests

XW, MS, SBG, ZS, VN, WLL, TCS and JRH declare that they have no competing interests. MPG and JNI are named inventors (along with the Mayo Clinic) in regard to non-provisional patent applications regarding tamoxifen and *CYP2D6*; the technology is not licensed, and no royalties have accrued.

## Authors' contributions

XW, MS, WLL, MPG, JNI, TCS and JRH conceived of the study, participated in its design and drafted the manuscript. XW, SBG, VN and JRH performed the laboratory experiments and analyzed the data. ZS performed the microarray and biological pathway analysis. All authors read and approved the final manuscript.

## Supplementary Material

Additional file 1 **Table indicating the sequences of primers used throughout this manuscript**. Click here for file

Additional file 2 **Confirmation of ERβ negativity in parental MCF7 cells**. **(A) **Real-time PCR and **(B) **Western blot analysis demonstrating that ERβ expression at both the mRNA and protein level is undetectable in parental MCF7 cells. These data are shown in comparison to one of the ERβ-expressing clonal cell lines (cell line #3). Click here for file

Additional file 3 **Table indicating the genes determined to be significantly regulated by 1 nM estrogen in parental and ERβ expressing MCF7 cells**. Click here for file

Additional file 4 **Table indicating the genes determined to be significantly regulated by 1 nM estrogen + 40 nM Endoxifen in parental and ERβ expressing MCF7 cells**. Click here for file

## References

[B1] Crewe HK Lennard MS Tucker GT Woods FR Haddock RE The effect of selective serotonin re-uptake inhibitors on cytochrome P4502D6 (CYP2D6) activity in human liver microsomes. 1992 Br J Clin Pharmacol 2004 58 S744 747 discussion 748-750 10.1111/j.1365-2125.2004.02282.x 15595963 PMC1884667

[B2] Desta Z Ward BA Soukhova NV Flockhart DA Comprehensive evaluation of tamoxifen sequential biotransformation by the human cytochrome P450 system *in vitro*: prominent roles for CYP3A and CYP2D6 J Pharmacol Exp Ther 2004 310 1062 1075 10.1124/jpet.104.065607 15159443

[B3] Stearns V Johnson MD Rae JM Morocho A Novielli A Bhargava P Hayes DF Desta Z Flockhart DA Active tamoxifen metabolite plasma concentrations after coadministration of tamoxifen and the selective serotonin reuptake inhibitor paroxetine J Natl Cancer Inst 2003 95 1758 1764 14652237 10.1093/jnci/djg108

[B4] Borges S Desta Z Li L Skaar TC Ward BA Nguyen A Jin Y Storniolo AM Nikoloff DM Wu L Hillman G Hayes DF Stearns V Flockhart DA Quantitative effect of CYP2D6 genotype and inhibitors on tamoxifen metabolism: implication for optimization of breast cancer treatment Clin Pharmacol Ther 2006 80 61 74 10.1016/j.clpt.2006.03.013 16815318

[B5] Jin Y Desta Z Stearns V Ward B Ho H Lee KH Skaar T Storniolo AM Li L Araba A Blanchard R Nguyen A Ullmer L Hayden J Lemler S Weinshilboum RM Rae JM Hayes DF Flockhart DA CYP2D6 genotype, antidepressant use, and tamoxifen metabolism during adjuvant breast cancer treatment J Natl Cancer Inst 2005 97 30 39 10.1093/jnci/dji005 15632378

[B6] Bijl MJ van Schaik RH Lammers LA Hofman A Vulto AG van Gelder T Stricker BH Visser LE The CYP2D6*4 polymorphism affects breast cancer survival in tamoxifen users Breast Cancer Res Treat 2009 118 125 130 10.1007/s10549-008-0272-2 19189212

[B7] Goetz MP Knox SK Suman VJ Rae JM Safgren SL Ames MM Visscher DW Reynolds C Couch FJ Lingle WL Weinshilboum RM Fritcher EG Nibbe AM Desta Z Nguyen A Flockhart DA Perez EA Ingle JN The impact of cytochrome P450 2D6 metabolism in women receiving adjuvant tamoxifen Breast Cancer Res Treat 2007 101 113 121 10.1007/s10549-006-9428-0 17115111

[B8] Goetz MP Rae JM Suman VJ Safgren SL Ames MM Visscher DW Reynolds C Couch FJ Lingle WL Flockhart DA Desta Z Perez EA Ingle JN Pharmacogenetics of tamoxifen biotransformation is associated with clinical outcomes of efficacy and hot flashes J Clin Oncol 2005 23 9312 9318 10.1200/JCO.2005.03.3266 16361630

[B9] Kiyotani K Mushiroda T Sasa M Bando Y Sumitomo I Hosono N Kubo M Nakamura Y Zembutsu H Impact of CYP2D6*10 on recurrence-free survival in breast cancer patients receiving adjuvant tamoxifen therapy Cancer Sci 2008 99 995 999 10.1111/j.1349-7006.2008.00780.x 18294285 PMC11158376

[B10] Newman WG Hadfield KD Latif A Roberts SA Shenton A McHague C Lalloo F Howell S Evans DG Impaired tamoxifen metabolism reduces survival in familial breast cancer patients Clin Cancer Res 2008 14 5913 5918 10.1158/1078-0432.CCR-07-5235 18794105

[B11] Nowell SA Ahn J Rae JM Scheys JO Trovato A Sweeney C MacLeod SL Kadlubar FF Ambrosone CB Association of genetic variation in tamoxifen-metabolizing enzymes with overall survival and recurrence of disease in breast cancer patients Breast Cancer Res Treat 2005 91 249 258 10.1007/s10549-004-7751-x 15952058

[B12] Schroth W Antoniadou L Fritz P Schwab M Muerdter T Zanger UM Simon W Eichelbaum M Brauch H Breast cancer treatment outcome with adjuvant tamoxifen relative to patient CYP2D6 and CYP2C19 genotypes J Clin Oncol 2007 25 5187 5193 10.1200/JCO.2007.12.2705 18024866

[B13] Wegman P Elingarami S Carstensen J Stal O Nordenskjold B Wingren S Genetic variants of CYP3A5, CYP2D6, SULT1A1, UGT2B15 and tamoxifen response in postmenopausal patients with breast cancer Breast Cancer Res 2007 9 R7 10.1186/bcr1640 17244352 PMC1851378

[B14] Xu Y Sun Y Yao L Shi L Wu Y Ouyang T Li J Wang T Fan Z Fan T Lin B He L Li P Xie Y Association between CYP2D6 *10 genotype and survival of breast cancer patients receiving tamoxifen treatment Ann Oncol 2008 19 1423 1429 10.1093/annonc/mdn155 18407954

[B15] Ramon y Cajal T Altes A Pare L del Rio E Alonso C Barnadas A Baiget M Impact of CYP2D6 polymorphisms in tamoxifen adjuvant breast cancer treatment Breast Cancer Res Treat 119 33 38 10.1007/s10549-009-0328-y 19189210

[B16] Wu X Hawse JR Subramaniam M Goetz MP Ingle JN Spelsberg TC The tamoxifen metabolite, endoxifen, is a potent antiestrogen that targets estrogen receptor alpha for degradation in breast cancer cells Cancer Res 2009 69 1722 1727 10.1158/0008-5472.CAN-08-3933 19244106

[B17] Waters KM Rickard DJ Riggs BL Khosla S Katzenellenbogen JA Katzenellenbogen BS Moore J Spelsberg TC Estrogen regulation of human osteoblast function is determined by the stage of differentiation and the estrogen receptor isoform J Cell Biochem 2001 83 448 462 10.1002/jcb.1242 11596113

[B18] Rickard DJ Waters KM Ruesink TJ Khosla S Katzenellenbogen JA Katzenellenbogen BS Riggs BL Spelsberg TC Estrogen receptor isoform-specific induction of progesterone receptors in human osteoblasts J Bone Miner Res 2002 17 580 592 10.1359/jbmr.2002.17.4.580 11918216

[B19] Monroe DG Getz BJ Johnsen SA Riggs BL Khosla S Spelsberg TC Estrogen receptor isoform-specific regulation of endogenous gene expression in human osteoblastic cell lines expressing either ERalpha or ERbeta J Cell Biochem 2003 90 315 326 10.1002/jcb.10633 14505348

[B20] Monroe DG Secreto FJ Subramaniam M Getz BJ Khosla S Spelsberg TC Estrogen receptor alpha and beta heterodimers exert unique effects on estrogen- and tamoxifen-dependent gene expression in human U2OS osteosarcoma cells Mol Endocrinol 2005 19 1555 1568 10.1210/me.2004-0381 15802376

[B21] Secreto FJ Monroe DG Dutta S Ingle JN Spelsberg TC Estrogen receptor alpha/beta isoforms, but not betacx, modulate unique patterns of gene expression and cell proliferation in Hs578T cells J Cell Biochem 2007 101 1125 1147 10.1002/jcb.21205 17520659

[B22] Kian Tee M Rogatsky I Tzagarakis-Foster C Cvoro A An J Christy RJ Yamamoto KR Leitman DC Estradiol and selective estrogen receptor modulators differentially regulate target genes with estrogen receptors alpha and beta Mol Biol Cell 2004 15 1262 1272 10.1091/mbc.E03-06-0360 14699072 PMC363122

[B23] Stossi F Barnett DH Frasor J Komm B Lyttle CR Katzenellenbogen BS Transcriptional profiling of estrogen-regulated gene expression via estrogen receptor (ER) alpha or ERbeta in human osteosarcoma cells: distinct and common target genes for these receptors Endocrinology 2004 145 3473 3486 10.1210/en.2003-1682 15033914

[B24] Lazennec G Bresson D Lucas A Chauveau C Vignon F ER beta inhibits proliferation and invasion of breast cancer cells Endocrinology 2001 142 4120 4130 10.1210/en.142.9.4120 11517191 PMC2040491

[B25] Thomas CG Strom A Lindberg K Gustafsson JA Estrogen receptor beta decreases survival of p53-defective cancer cells after DNA damage by impairing G(2)/M checkpoint signaling Breast Cancer Res Treat 2010 in press 20623183 10.1007/s10549-010-1011-z

[B26] Sotoca AM van den Berg H Vervoort J van der Saag P Strom A Gustafsson JA Rietjens I Murk AJ Influence of cellular ERalpha/ERbeta ratio on the ERalpha-agonist induced proliferation of human T47 D breast cancer cells Toxicol Sci 2008 105 303 311 10.1093/toxsci/kfn141 18644836 PMC2527638

[B27] Paruthiyil S Parmar H Kerekatte V Cunha GR Firestone GL Leitman DC Estrogen receptor beta inhibits human breast cancer cell proliferation and tumor formation by causing a G2 cell cycle arrest Cancer Res 2004 64 423 428 10.1158/0008-5472.CAN-03-2446 14729654

[B28] Strom A Hartman J Foster JS Kietz S Wimalasena J Gustafsson JA Estrogen receptor beta inhibits 17beta-estradiol-stimulated proliferation of the breast cancer cell line T47D Proc Natl Acad Sci USA 2004 101 1566 1571 10.1073/pnas.0308319100 14745018 PMC341775

[B29] Esslimani-Sahla M Simony-Lafontaine J Kramar A Lavaill R Mollevi C Warner M Gustafsson JA Rochefort H Estrogen receptor beta (ER beta) level but not its ER beta cx variant helps to predict tamoxifen resistance in breast cancer Clin Cancer Res 2004 10 5769 5776 10.1158/1078-0432.CCR-04-0389 15355905

[B30] Fleming FJ Hill AD McDermott EW O'Higgins NJ Young LS Differential recruitment of coregulator proteins steroid receptor coactivator-1 and silencing mediator for retinoid and thyroid receptors to the estrogen receptor-estrogen response element by beta-estradiol and 4-hydroxytamoxifen in human breast cancer J Clin Endocrinol Metab 2004 89 375 383 10.1210/jc.2003-031048 14715875

[B31] Hopp TA Weiss HL Parra IS Cui Y Osborne CK Fuqua SA Low levels of estrogen receptor beta protein predict resistance to tamoxifen therapy in breast cancer Clin Cancer Res 2004 10 7490 7499 10.1158/1078-0432.CCR-04-1114 15569979

[B32] Iwase H Zhang Z Omoto Y Sugiura H Yamashita H Toyama T Iwata H Kobayashi S Clinical significance of the expression of estrogen receptors alpha and beta for endocrine therapy of breast cancer Cancer Chemother Pharmacol 2003 52 Suppl 1 S34 38 10.1007/s00280-003-0592-1 12819932

[B33] Mann S Laucirica R Carlson N Younes PS Ali N Younes A Li Y Younes M Estrogen receptor beta expression in invasive breast cancer Hum Pathol 2001 32 113 118 10.1053/hupa.2001.21506 11172304

[B34] Murphy LC Leygue E Niu Y Snell L Ho SM Watson PH Relationship of coregulator and oestrogen receptor isoform expression to de novo tamoxifen resistance in human breast cancer Br J Cancer 2002 87 1411 1416 10.1038/sj.bjc.6600654 12454770 PMC2376286

[B35] Myers E Fleming FJ Crotty TB Kelly G McDermott EW O'Higgins N J Hill AD Young LS Inverse relationship between ER-beta and SRC-1 predicts outcome in endocrine-resistant breast cancer Br J Cancer 2004 91 1687 1693 15477868 10.1038/sj.bjc.6602156 PMC2409954

[B36] Nakopoulou L Lazaris AC Panayotopoulou EG Giannopoulou I Givalos N Markaki S Keramopoulos A The favourable prognostic value of oestrogen receptor beta immunohistochemical expression in breast cancer J Clin Pathol 2004 57 523 528 10.1136/jcp.2003.008599 15113861 PMC1770285

[B37] Omoto Y Inoue S Ogawa S Toyama T Yamashita H Muramatsu M Kobayashi S Iwase H Clinical value of the wild-type estrogen receptor beta expression in breast cancer Cancer Lett 2001 163 207 212 10.1016/S0304-3835(00)00680-7 11165756

[B38] Sugiura H Toyama T Hara Y Zhang Z Kobayashi S Fujii Y Iwase H Yamashita H Expression of estrogen receptor beta wild-type and its variant ERbetacx/beta2 is correlated with better prognosis in breast cancer Jpn J Clin Oncol 2007 37 820 828 10.1093/jjco/hym114 17932113

[B39] Miller WR Anderson TJ Dixon JM Saunders PT Oestrogen receptor beta and neoadjuvant therapy with tamoxifen: prediction of response and effects of treatment Br J Cancer 2006 94 1333 1338 10.1038/sj.bjc.6603082 16622466 PMC2361404

[B40] O'Neill PA Davies MP Shaaban AM Innes H Torevell A Sibson DR Foster CS Wild-type oestrogen receptor beta (ERbeta1) mRNA and protein expression in Tamoxifen-treated post-menopausal breast cancers Br J Cancer 2004 91 1694 1702 15477865 10.1038/sj.bjc.6602183 PMC2409946

[B41] Skliris GP Leygue E Curtis-Snell L Watson PH Murphy LC Expression of oestrogen receptor-beta in oestrogen receptor-alpha negative human breast tumours Br J Cancer 2006 95 616 626 10.1038/sj.bjc.6603295 16880783 PMC2360679

[B42] Jensen EV Cheng G Palmieri C Saji S Makela S Van Noorden S Wahlstrom T Warner M Coombes RC Gustafsson JA Estrogen receptors and proliferation markers in primary and recurrent breast cancer Proc Natl Acad Sci USA 2001 98 15197 15202 10.1073/pnas.211556298 11734621 PMC65006

[B43] Speirs V Parkes AT Kerin MJ Walton DS Carleton PJ Fox JN Atkin SL Coexpression of estrogen receptor alpha and beta: poor prognostic factors in human breast cancer? Cancer Res 1999 59 525 528 9973193

[B44] Honma N Horii R Iwase T Saji S Younes M Takubo K Matsuura M Ito Y Akiyama F Sakamoto G Clinical importance of estrogen receptor-beta evaluation in breast cancer patients treated with adjuvant tamoxifen therapy J Clin Oncol 2008 26 3727 3734 10.1200/JCO.2007.14.2968 18669459

[B45] Poola I Fuqua SA De Witty RL Abraham J Marshallack JJ Liu A Estrogen receptor alpha-negative breast cancer tissues express significant levels of estrogen-independent transcription factors, ERbeta1 and ERbeta5: potential molecular targets for chemoprevention Clin Cancer Res 2005 11 7579 7585 10.1158/1078-0432.CCR-05-0728 16243834

[B46] Shaaban AM Green AR Karthik S Alizadeh Y Hughes TA Harkins L Ellis IO Robertson JF Paish EC Saunders PT Groome NP Speirs V Nuclear and cytoplasmic expression of ERbeta1, ERbeta2, and ERbeta5 identifies distinct prognostic outcome for breast cancer patients Clin Cancer Res 2008 14 5228 5235 10.1158/1078-0432.CCR-07-4528 18698041

[B47] Primer3 Input (v.0.4.0) http://frodo.wi.mit.edu/primer3/

[B48] Ballman KV Grill DE Oberg AL Therneau TM Faster cyclic loess: normalizing RNA arrays via linear models Bioinformatics 2004 20 2778 2786 10.1093/bioinformatics/bth327 15166021

[B49] Gene Expression Omnibus (GEO) http://www.ncbi.nlm.nih.gov/geo/

[B50] Meyers MJ Sun J Carlson KE Marriner GA Katzenellenbogen BS Katzenellenbogen JA Estrogen receptor-beta potency-selective ligands: structure-activity relationship studies of diarylpropionitriles and their acetylene and polar analogues J Med Chem 2001 44 4230 4251 10.1021/jm010254a 11708925

[B51] Iwao K Miyoshi Y Egawa C Ikeda N Noguchi S Quantitative analysis of estrogen receptor-beta mRNA and its variants in human breast cancers Int J Cancer 2000 88 733 736 10.1002/1097-0215(20001201)88:5<733::AID-IJC8>3.0.CO;2-M 11072241

[B52] Leygue E Dotzlaw H Watson PH Murphy LC Altered estrogen receptor alpha and beta messenger RNA expression during human breast tumorigenesis Cancer Res 1998 58 3197 3201 9699641

[B53] Leygue E Dotzlaw H Watson PH Murphy LC Expression of estrogen receptor beta1, beta2, and beta5 messenger RNAs in human breast tissue Cancer Res 1999 59 1175 1179 10096542

[B54] Zhao C Lam EW Sunters A Enmark E De Bella MT Coombes RC Gustafsson JA Dahlman-Wright K Expression of estrogen receptor beta isoforms in normal breast epithelial cells and breast cancer: regulation by methylation Oncogene 2003 22 7600 7606 10.1038/sj.onc.1207100 14576822

[B55] Hodges-Gallagher L Valentine CD El Bader S Kushner PJ Estrogen receptor beta increases the efficacy of antiestrogens by effects on apoptosis and cell cycling in breast cancer cells Breast Cancer Res Treat 2008 109 241 250 10.1007/s10549-007-9640-6 17638070

[B56] Chang EC Frasor J Komm B Katzenellenbogen BS Impact of estrogen receptor beta on gene networks regulated by estrogen receptor alpha in breast cancer cells Endocrinology 2006 147 4831 4842 10.1210/en.2006-0563 16809442

[B57] Liu Y Gao H Marstrand TT Strom A Valen E Sandelin A Gustafsson JA Dahlman-Wright K The genome landscape of ERalpha- and ERbeta-binding DNA regions Proc Natl Acad Sci USA 2008 105 2604 2609 10.1073/pnas.0712085105 18272478 PMC2268183

[B58] Powell E Xu W Intermolecular interactions identify ligand-selective activity of estrogen receptor alpha/beta dimers Proc Natl Acad Sci USA 2008 105 19012 19017 10.1073/pnas.0807274105 19022902 PMC2596243

[B59] Ju YH Allred KF Allred CD Helferich WG Genistein stimulates growth of human breast cancer cells in a novel, postmenopausal animal model, with low plasma estradiol concentrations Carcinogenesis 2006 27 1292 1299 10.1093/carcin/bgi370 16537557

[B60] Peeters PH Keinan-Boker L van der Schouw YT Grobbee DE Phytoestrogens and breast cancer risk. Review of the epidemiological evidence Breast Cancer Res Treat 2003 77 171 183 10.1023/A:1021381101632 12602916

[B61] Mersereau JE Levy N Staub RE Baggett S Zogovic T Chow S Ricke WA Tagliaferri M Cohen I Bjeldanes LF Leitman DC Liquiritigenin is a plant-derived highly selective estrogen receptor beta agonist Mol Cell Endocrinol 2008 283 49 57 10.1016/j.mce.2007.11.020 18177995 PMC2277338

[B62] Saunders PT Millar MR Williams K Macpherson S Bayne C O'Sullivan C Anderson TJ Groome NP Miller WR Expression of oestrogen receptor beta (ERbeta1) protein in human breast cancer biopsies Br J Cancer 2002 86 250 256 10.1038/sj.bjc.6600035 11870515 PMC2375186

[B63] Skliris GP Carder PJ Lansdown MR Speirs V Immunohistochemical detection of ERbeta in breast cancer: towards more detailed receptor profiling? Br J Cancer 2001 84 1095 1098 10.1054/bjoc.2001.1721 11308260 PMC2363860

[B64] Fuqua SA Schiff R Parra I Moore JT Mohsin SK Osborne CK Clark GM Allred DC Estrogen receptor beta protein in human breast cancer: correlation with clinical tumor parameters Cancer Res 2003 63 2434 2439 12750263 PMC4482102

[B65] Skliris GP Leygue E Watson PH Murphy LC Estrogen receptor alpha negative breast cancer patients: estrogen receptor beta as a therapeutic target J Steroid Biochem Mol Biol 2008 109 1 10 10.1016/j.jsbmb.2007.12.010 18243688

