# Supplementary figures and images for: Estrogen receptor-beta sensitizes breast cancer cells to the anti-estrogenic actions of endoxifen
Source: Breast Cancer Res. 2011 Mar 10;13(2):R27. doi: 10.1186/bcr2844 (PMC3219188; doi:10.1186/bcr2844)

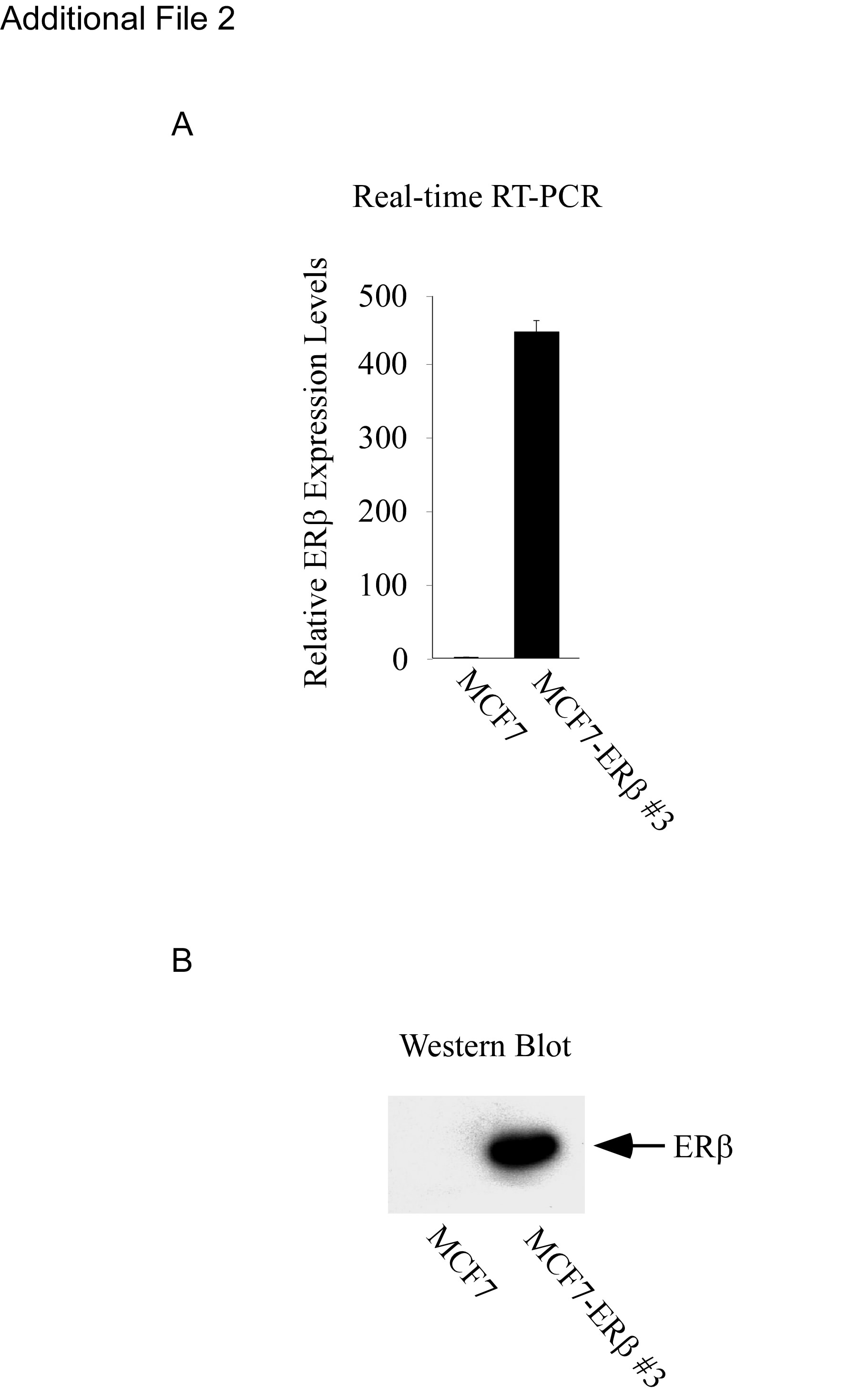

Supplement: Additional file 2 — Confirmation of ERβ negativity in parental MCF7 cells. (A) Real-time PCR and (B) Western blot analysis demonstrating that ERβ expression at both the mRNA and protein level is undetectable in parental MCF7 cells. These data are shown in comparison to one of the ERβ-expressing clonal cell lines (cell line #3). [file bcr2844-S2.JPEG]
